# Hybrid Convolutional Neural Network for Localization of Epileptic Focus Based on iEEG

**DOI:** 10.1155/2021/6644365

**Published:** 2021-04-27

**Authors:** Linfeng Sui, Xuyang Zhao, Qibin Zhao, Toshihisa Tanaka, Jianting Cao

**Affiliations:** ^1^Graduate School of Engineering, Saitama Institute of Technology, 369-0293, Japan; ^2^RIKEN Center for Advanced Intelligence Project (AIP), 103-0027, Japan; ^3^Department of Electrical and Electronic Engineering, Tokyo University of Agriculture and Technology, 184-8588, Japan

## Abstract

Epileptic focus localization by analysing intracranial electroencephalogram (iEEG) plays a critical role in successful surgical therapy of resection of the epileptogenic lesion. However, manual analysis and classification of the iEEG signal by clinicians are arduous and time-consuming and excessively depend on the experience. Due to individual differences of patients, the iEEG signal from different patients usually shows very diverse features even if the features belong to the same class. Accordingly, automatic detection of epileptic focus is required to improve the accuracy and to shorten the time for treatment. In this paper, we propose a novel feature fusion-based iEEG classification method, a deep learning model termed Time-Frequency Hybrid Network (TF-HybridNet), in which short-time Fourier transform (STFT) and 1d convolution layers are performed on the input iEEG in parallel to extract features of the time-frequency domain and feature maps. And then, the time-frequency features and feature maps are fused and fed to a 2d convolutional neural network (CNN). We used the Bern-Barcelona iEEG dataset for evaluating the performance of TF-HybridNet, and the experimental results show that our approach is able to differentiate the focal from nonfocal iEEG signal with an average classification accuracy of 94.3% and demonstrates an improved accuracy rate compared to the model using only STFT or one-dimensional convolutional layers as feature extraction.

## 1. Introduction

Epilepsy is a chronic disease of the brain, and it is characterized by recurrent and unpredictable seizures, which are brief episodes of involuntary movements and even accompanied by transient loss of consciousness [1]. Currently, with approximately 50 million epilepsy sufferers worldwide according to the World Health Organization (WHO), epilepsy is one of the most common neurological diseases globally, making it a significant challenge for healthcare and social services [1]. The cause of seizure episodes is excessive electrical discharging in a group of brain neurons. Electroencephalography is therefore a commonly used method to measure brain activity through the recording of electrical activity and has been widely used for the diagnosis and treatment of varied neurological conditions such as brain death, epilepsy, Alzheimer's, and coma [2]. Considering it is uncertain that symptoms will present in the iEEG signal at all times, and iEEG should be monitored and recorded in the long term. During this process, huge amounts of data are generated and experienced neurological experts subsequently analyse abnormalities in brain activities via visual inspection. This task is time-consuming that could lead to a serious delay of days or even weeks of treatment. In recent years, various automatic diagnostic methods have been proposed to assist neurologists by accelerating the interpreting process, thereby reducing workload [3, 4]. Current methods mainly focused on tasks such as seizure detection, seizure prediction, and seizure type classification. Statistics show that up to 70% of patients could be successfully treated with the proper use of antiepileptic drugs (AEDs) [1]. Nevertheless, for patients who respond poorly to drug treatments, resection surgery for epileptogenic tissues might be one of the most promising treatments in controlling epileptic seizures. Hence, it is crucial to determine the seizure area in surgical therapy, and there is a very strong demand for the automatic detection of epileptic focus localization. iEEG is recorded directly from the cerebral cortex, and iEEG signals recorded from the epileptogenic area are more stationary and less random than iEEG signals recorded from the normal area [5]. This nature makes it enable to be used for identification of location effectively. The task of seizure focus localization is not largely developed owing to factors such as the complex nature of the task and rare clinical datasets [6]. The mainly used dataset is the publicly available Bern-Barcelona iEEG dataset, which was collected by Andrzejak et al. at the Department of Neurology of the University of Bern [7].

In recent years, various automatic focus detection methods through the classifying iEEG signal into focal and nonfocal have been proposed [8]. Most of those are usually divided into three main steps, preprocessing, feature extraction, and classification. In the preprocessing step, various filtration or normalization is applied to the raw signal. In the feature extraction step, to extract the most discriminative features, commonly used methods include empirical mode decomposition (EMD) [9], entropy, and time-frequency analysis methods like Hilbert-Huang transform, Fourier transform (FT) [10], STFT [11], and wavelet transforms (WT) [12, 13]. Particularly, STFT has been established that it is suitable for iEEG signal processing by extracting time-frequency domain features [14]. In the classification step, support vector machines (SVM) [9], logistic regression (LR) [15], and *K*-Nearest Neighbor (KNN) method [16] are usually be used. With the rapid development of deep learning models, automatic feature-based approaches have been successfully applied to classification problems [17]; in particular, CNN is regarded as one of the most successful and widely used deep learning models. In our previous research work [18, 19], two individual feature extraction methods, the Time-Frequency Convolutional Neural Network (TFCNN) and the Mixed-CNN, were proposed and have proven to be effective. The main contribution of this paper is that we propose TF-HybridNet, a deep learning model to diversify features by combining time-frequency analysis and learnable automatic feature extraction methods. We compare the TF-HybridNet accuracy with the TFCNN and Mixed-CNN, and experiments show that the multifeature extraction method produces higher iEEG classification accuracy compared to the individual feature extraction method. The proposed framework does not only have strong feature learning capabilities but also have adaptive iEEG features for higher classification performance without much human intervention.

The rest of the article is organized as follows: Section 2 describes the dataset used in the experiment and the method of CNN and STFT.  Section 3 describes a comparison of the architecture of three deep learning models proposed. The experimental results are presented in Section 4, and the last is the conclusion of this paper.

## 2. Materials and Methods

In this section, we firstly introduce the Bern-Barcelona iEEG dataset. Then, we discuss the STFT, a powerful general-purpose tool for time-frequency domain feature extraction. The working principles used in this paper are described at last, including a 1d convolutional layer, 2d convolutional layer, and various neural network components. This collection of working principles and components provides an essential basis for the three deep learning models proposed in Section 3.

### 2.1. Dataset

**Figure 1:**
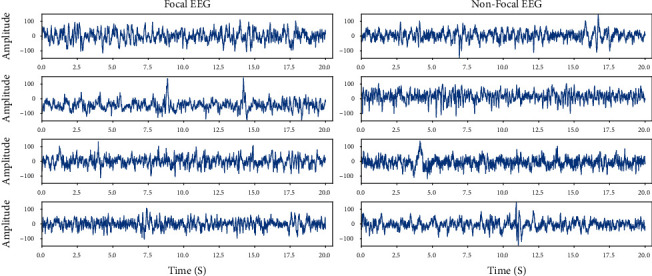
An example of the focal and nonfocal iEEG signal.

The Bern-Barcelona iEEG dataset was recorded from five patients suffering from long-standing drug-resistant temporal lobe epilepsy which were candidates for surgery. The signal recorded from the focus region (lesion) was labeled as the focal signal; otherwise, the signal was labeled as the nonfocal signal. The dataset contains 3750 focal iEEG signal pairs and 3750 nonfocal iEEG signal pairs. Each pair of iEEG signal from adjacent channels was sampled for 20 seconds at a frequency of 512 Hz and was band-pass filtered between 0.5 and 150 Hz with a fourth-order Butterworth filter. The iEEG signal recorded during the seizure and three hours after the last seizure was excluded to guarantee to discard the seizure iEEG signal. An example of the focal and nonfocal iEEG signal is shown in Figure 1, respectively.

### 2.2. Short-Time Fourier Transform

On account of the instability of the iEEG signal, it is extremely difficult to extract the key features by some commonly used time-frequency analysis methods such as Fourier transform [20]. The STFT, as a Fourier-related transform, is used to equally divide the raw signal into shorter segments of length by a window function which is nonzero for only a short period of time, so that the segments of the signal are approximately stationary. The Fourier transform of the shorter segments is computed as the window function is sliding along the time axis, obtaining the spectrum, a 2-dimensional representation of the signal. Hence, it is demonstrated that the time-frequency domain features extracted by STFT are suitable for classifying iEEG signal of epilepsy [20]. For a determined signal *x*(*t*), the time-frequency domain at each time point can be obtained by

(1) $$\begin{array}{c} \text{STFT}\left\{x\left(t\right)\right\}\left(\tau ,\omega \right)=\int_{-\infty }^{\infty }x\left(t\right)w\left(t-\tau \right)e^{-j\omega t}dt, \end{array}$$

where *w*(*t*) is the Hann window function centered around zero.

**Figure 2:**
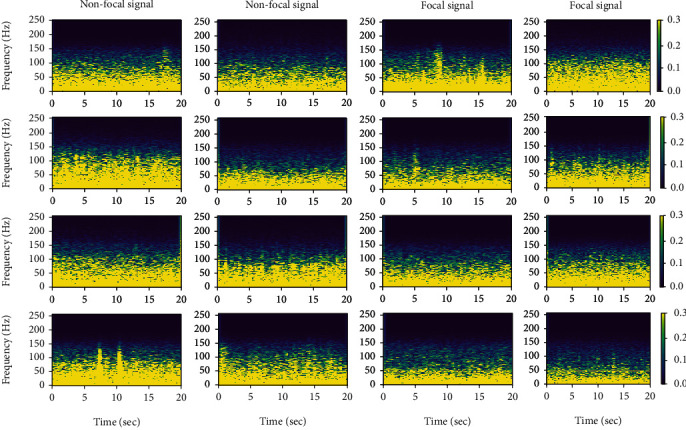
STFT of the focal and nonfocal iEEG signal.

Examples of the spectrogram of the iEEG signal (focal and nonfocal) are shown in Figure 2.

### 2.3. Convolutional Neural Network

CNN is a subset of deep learning which has recently been successfully used in numerous tasks in different research fields of images and time series classification (TSC), such as biomedical imaging, iEEG/Electrocardiography (ECG) signal, and motion sensor data and speech. The CNN model consists of an input and an output layer, as well as multiple hidden layers, and the early layers following input layers are general convolutional layers. Convolution is a mathematical operation that is used to extract the feature map by sliding the convolution kernel over the input data, which helps extract particular features.

#### 2.3.1. One-Dimensional Convolutional Layer

The overlapping values of the kernel and the input vector for each position the kernel is sliding are multiplied together, and the sum of the results will be the value of the feature map at the point on the input vector where it corresponds to the midpoint of the kernel. For an input vector *f* with length *n* and a kernel *g* with length *m*, *f*∗*g* is the convolution of *f* and *g* and is defined as

(2) $$\begin{array}{c} \left(f*g\right)\left(x\right)=\overset{m}{\underset{u=1}{\sum }}g\left(u\right)\cdot f\left(x-u+\frac{m}{2}\right). \end{array}$$

#### 2.3.2. Two-Dimensional Convolutional Layer

For 2d convolution, just as 1d convolution, we slide the 2d kernel over each pixel of the input image and then multiply the corresponding entries of the input image and kernel; the sum of the results will be the value of the feature map. The activation map is obtained by computing the dot product of the input file and the filter. Then, after additive bias and a nonlinear map by activation functions, feature maps of the convolutional layer are output to feed to the next layer in the CNN model.

#### 2.3.3. Pooling Layer

After the convolution layer, feature maps are usually passed to the pooling layer and different from the convolution operation; pooling has no parameters. In the pooling layer, the feature maps are separated into many rectangle regions, and then, each region feature is obtained. It enables downsampling each feature map independently to reduce the dimensionality, lower the calculation complexity, and prevent overfitting. Various pooling operations, for instance, max pooling operation, select only the maximum value in the pooling window, while mean pooling obtains the mean value of the pooling window.

#### 2.3.4. Batch Normalization Layer

The batch normalization layer is applied to normalize the output feature map obtained from the previous layer by subtracting the batch mean and dividing by batch standard deviation, to fight the internal covariate shift problem and increase the stability of neural networks. For the input *x* obtained from the previous layer, the batch normalization layer first calculates the mean *μ*_*ℬ*_ and variance *σ*_*ℬ*_^2^ of a minibatch *ℬ* of size *m* by equations (3) and (4). Then, normalized values *x*_*i*_ are calculated as equation (5) where *ε* is a constant added to the minibatch variance for numerical stability. Finally, the *x*_*i*_ are shifted and scaled as equation (6) that the parameters *γ* and *β* are to be learned.

(3) $$\begin{array}{c} \mu_{B}=\frac{1}{m}\overset{m}{\underset{i=1}{\sum }}x_{i}, \end{array}$$

(4) $$\begin{array}{c} \sigma_{B}^{2}=\frac{1}{m}\overset{m}{\underset{i=1}{\sum }}{\left(x_{i}-\mu_{B}\right)}^{2}, \end{array}$$

(5) $$\begin{array}{c} \overset{¯}{x_{i}}=\frac{x_{i}-\mu_{B}}{\sqrt{\sigma_{B}^{2}+\varepsilon }}, \end{array}$$

(6) $$\begin{array}{c} y_{i}=\gamma \overset{¯}{x_{i}}+\beta . \end{array}$$

#### 2.3.5. Fully Connected Layer

In the fully connected layer, all the 2d feature maps from the upper layer are represented by a one-dimensional feature vector as the input of this layer. In this paper, the output is obtained by doing dot products between the feature vector and learnable weight vector, adding learnable bias and then responding to the activation function.

## 3. Neural Network Architecture

Conventional CNNs are hierarchical architectures based on an alternation of convolutional layers with pooling layers and batch normalization layers and followed by a fully connected layer.

### 3.1. Time-Frequency Convolutional Neural Network

**Figure 3:**
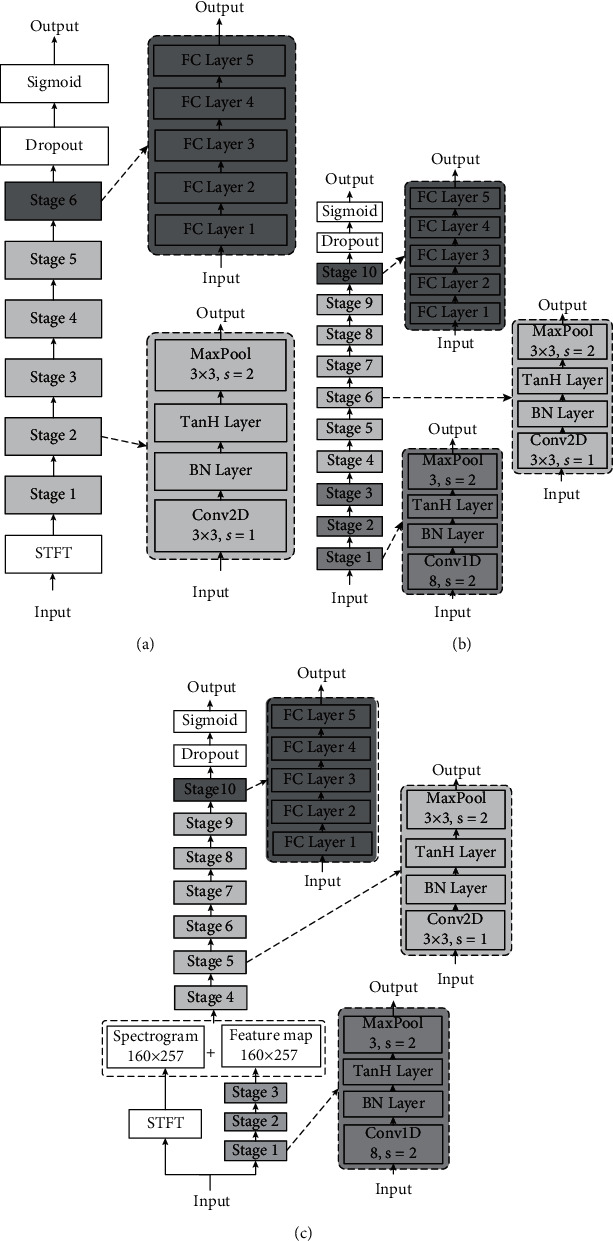
An overview of architectures of TFCNN (a), Mixed-CNN (b), and TF-HybridNet (c).

In our previous research, we proposed an architecture that combines time-frequency analysis and a two-dimensional convolutional neural network. A TFCNN network consists of an STFT layer, five subsequent stages, five FC stages, a dropout layer, and a final output layer, which is illustrated in Figure 3(a).

In that architecture, the iEEG signal is firstly transformed by the STFT layer to extract local features individually based on the local correlation among the time-frequency domain. Then, discriminative features that are built by connecting the local features are learned, and classification is performed by the TFCNN. The specific training process is as follows: the time-frequency spectrogram with size 257 × 101 is firstly convoluted by using a 3 × 3 filter by sliding with stride 1 and set 10 channels to feature map, and each feature map has the same size as the input spectrogram. Then, batch normalization (BN) and max pooling operation are successively implemented in the batch normalization layer and max pooling layer. And these two steps are repeated 5 times, except that the size of input and output is decided by the former layer, and channels of the feature map increase exponentially.

### 3.2. Mixed Convolutional Neural Network

In the previous TFCNN architecture, before feeding into the neural network, the signal needs to perform extraction and selection of features manually. The most used time-frequency analysis method like STFT has the capability to extract local information at a one-time scale determined by a single filter, limiting the flexibility of the model. To address this problem, considering a convolution can be seen as applying and sliding a filter over the time series; instead of the STFT, we use 1d convolution layers in the earlier layers. It is easier to optimize the parameter configuration when each layer is treated independently, and it also enables using different input feature maps or receptive field sizes. A Mixed-CNN consists of eight convolution stages, five FC stages, a dropout layer, and a final output layer, which is illustrated in Figure 3(b). From stages 1 to 3, each stage begins with an 8 × 1 1d convolution layer with a stride of 2, which is then followed by the BN layer and 3 × 1 max pooling layer also with a stride of 2. The size of the output feature map of stage 3 is 159 × 256. The feature maps from 1d convolution layers are reshaped and then successively fed to subsequent five 2d convolution stages and fully connected layer to perform further feature extraction and classification.

### 3.3. Time-Frequency Hybrid Network

Inspired by the performances of the previous two models, we propose a hybrid model combining time-frequency analysis and Mixed-CNN. As shown in the architecture of TF-HybridNet in Figure 3(c), before feeding into 2d convolution layers to perform further feature extraction, the spectrogram obtained from STFT and the feature map obtained from 1d convolutional layers are both adjusted into a size of 160 × 257 and are stacked together in sequence depthwise. The remaining parts of the model are almost the same as Mixed-CNN.

## 4. Results and Discussion

The proposed models were implemented on a workstation with 12 Intel Core i7 3.50 GHz (5930 K), a GeForce RTX 2080 Ti graphics processing unit (GPU), and 128 GB random-access memory (RAM) using the Python programming language on the TensorFlow framework. The 5-fold cross-validation and he 10-fold cross-validation are used in this paper. In 5-fold cross-validation, 60% of the dataset is used as the training set and 20% is used as a validation set while the remaining 20% is used as the test set. And in 10-fold cross-validation, the distribution proportion is set to 80%, 10%, and 10%. It requires a lot of computational overhead to use one iteration of the full training set to perform each epoch; therefore, in each epoch of the training, the training set is randomly divided into 100, 120, and 200 batches separately in TFCNN, Mixed-CNN, and TF-HybridNet, which are fed into the network in turn.

**Figure 4:**
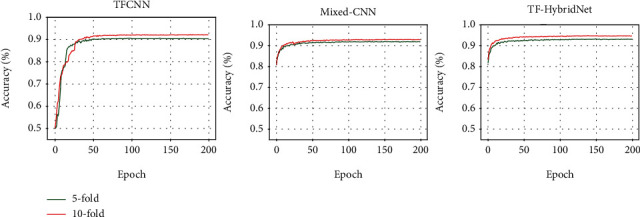
The accuracy of the validation set on TFCNN, Mixed-CNN, and TF-HybridNet.

The training performance of the model was monitored during the training stage until getting the best accuracy on the training set with minimum train loss. And we validate the networks by using a validation set after each epoch of training. The accuracy of the validation set across classification by the different models is shown in Figure 4. It can be seen that there is no overfitting problem in the three models; during the training period, the validation accuracy is steady at the end of the training. Finally, for performance evaluation of the trained models with the test set, we have selected various evaluation criteria, including accuracy, precision, recall, Matthews correlation coefficient (MCC), and kappa score *κ* which can be calculated by

(7) $$\begin{array}{c} \text{Accuracy}=\frac{\text{TP}+\text{TN}}{\text{TP}+\text{FP}+\text{FN}+\text{TN}},\\ \text{Precision}=\frac{\text{TP}}{\text{TP}+\text{FP}},\\ \text{Recall}=\frac{\text{TP}}{\text{TP}+\text{FN}},\\ \text{MCC}=\frac{\text{TP}\times \text{TN}-\text{TP}\times \text{FN}}{\sqrt{\left(\text{TP}+\text{FP}\right)\times \left(\text{TP}+\text{FN}\right)\times \left(\text{TN}+\text{FP}\right)\times \left(\text{TN}+\text{FN}\right)}},\\ \kappa =\frac{\rho_{o}-\rho_{e}}{1-\rho_{e}},\text{where }\rho_{o}=\frac{\text{TP}+\text{TN}}{\text{TP}+\text{FP}+\text{FN}+\text{TN}},\rho_{e}=\frac{\left(\text{TP}+\text{FN}\right)\times \left(\text{TP}+\text{FP}\right)+\left(\text{FP}+\text{TN}\right)\times \left(\text{FN}+\text{TN}\right)}{{\left(\text{TP}+\text{FP}+\text{FN}+\text{TN}\right)}^{2}}, \end{array}$$

The abbreviations represent true positive (TP), false positive (FP), true negative (TN), and false negative (FN).

Confusion matrices and performance measures through 5-fold/10-fold cross-validation obtained for TFCNN, Mixed-CNN, and TF-HybridNet are shown in Table 1. Our results show that the developed TF-HybridNet model performed better than the other two models both during the training and testing periods. And considering that the result of 10-fold cross-validation is better than that of 5-fold cross-validation, the performance could be improved with more numbers of iEEG data. Compared with other published state-of-the-art methods shown in Table 1, the proposed TF-HybridNet managed to obtain 94.3% accuracy. And the advantage of this method is that it is less signal preprocessing for feature extraction and selection.

## 5. Conclusions

Since the manual visual inspection of iEEG is a time-consuming process, automation of the detection of epileptic focus by an effective classifier will have the potential to solve the delay issue in treatment. In the case of signal feature extraction, STFT may be able to extract some but not all specific features of the time-frequency domain and the convolutional layer is similarly able to extract partial latent features of iEEG. In addition, iEEG signal from different patients usually shows very diverse features due to individual differences of patients, even if the features belong to the same class. It leads to that each feature extraction methods usually obtain different results on different datasets in the signal processing field. To solve these problems, we propose to adopt a feature fusion-based iEEG classification method which can make up for the shortage of traditional feature extraction and deep learning techniques. In this paper, we present and compare the performance of three different models (TFCNN, Mixed-CNN, and TF-HybridNet) for iEEG signal classification as the focal and nonfocal iEEG signal. Among the three models, the TF-HybridNet model performs the best result both 5-fold and 10-fold. Even though this proposed model could not yield the best classification performance as compared to the published works shown in Table 2, the proposed TF-HybridNet model still managed to obtain 94.3% accuracy. This shows that the TF-HybridNet is effective with much efficiency and timesaving to assist neurological clinicians to detect the focal epileptic seizure area.

## Figures and Tables

**Table:** (a) TFCNN model

		Focal	Nonfocal	Precision	Recall	Accuracy	Kappa score	MCC
5-fold	Focal	TP = 6829	FN = 671	90.4%	91.1%	90.7%	0.814	0.814
	Nonfocal	FP = 725	TN = 6775					

10-fold	Focal	TP = 6921	FN = 579	91.6%	92.3%	91.9%	0.838	0.838
	Nonfocal	FP = 633	TN = 6867					

**Table:** (b) Mixed-CNN model

		Focal	Nonfocal	Precision	Recall	Accuracy	Kappa score	MCC
5-fold	Focal	TP = 6898	FN = 602	91.7%	92.0%	91.8%	0.837	0.837
	Nonfocal	FP = 622	TN = 6878					

10-fold	Focal	TP = 6948	FN = 552	92.3%	92.6%	92.5%	0.849	0.849
	Nonfocal	FP = 578	TN = 6922					

**Table:** (c) TF-HybridNet model

		Focal	Nonfocal	Precision	Recall	Accuracy	Kappa score	MCC
5-fold	Focal	TP = 7002	FN = 498	93.0%	93.4%	93.2%	0.864	0.864
	Nonfocal	FP = 523	TN = 6977					

10-fold	Focal	TP = 7075	FN = 425	94.3%	94.3%	94.3%	0.887	0.887
	Nonfocal	FP = 426	TN = 7074					

**Table 2:** Detection results of focal and nonfocal EEG signals of published journal articles using the Bern-Barcelona EEG database.

Author (year)	Feature extraction methods	Classifier	Accuracy
Sharma et al. (2015) [21]	EMD, entropy	SVM	87.0%
Sriraam et al. (2017) [22]	Statistical, frequency-based, entropy, FD, Wilcoxon test	SVM	92.2%
Sharma et al. (2017) [23]	WFB, entropy, *t*-test	LS-SVM	94.3%
Das and Bhuiyan (2016) [24]	EMD-DWT, entropy	KNN	89.4%
Bhattacharyya et al. (2017) [25]	EWT, RPS, CTM	LS-SVM	90.0%
Gupta et al. (2017) [26]	FAWT, entropy, Kruskal-Wallis test	LS-SVM	94.4%
Zhao et al. (2018) [27]	Entropy	CNN	83.0%
Daoud and Bayoumi (2020) [28]	DCAE	MLP	93.2%
TFCNN	STFT	2d-CNN	91.9%
Mixed-CNN	1d convolution layer	2d-CNN	92.5%
TF-HybridNet	STFT, 1d convolution layer	2d-CNN	94.3%

## Data Availability

The Bern-Barcelona EEG database: https://www.upf.edu/web/ntsa/downloads/-/asset_publisher/xvT6E4pczrBw/content/2012-nonrandomness-nonlinear-dependence-and-nonstationarity-of-electroencephalographic-recordings-from-epilepsy-patients?inheritRedirect=falseamp;redirect=https%3A%2F%2Fwww.upf.edu%2Fweb%2Fntsa%2Fdownloads%3Fp_p_id%3D101_INSTANCE_xvT6E4pczrBw%26p_p_lifecycle%3D0%26p_p_state%3Dnormal%26p_p_mode%3Dview%26p_p_col_id%3Dcolumn-1%26p_p_col_count%3D1#.X6ZxJkL7TX9.
